# A dataset from bottom trawl survey around Taiwan

**DOI:** 10.3897/zookeys.198.3032

**Published:** 2012-05-30

**Authors:** Kwang-Tsao Shao, Jack Lin, Chung-Han Wu, Hsin-Ming Yeh, Tun-Yuan Cheng

**Affiliations:** 1 Biodiversity Research Center, Academia Sinica, No. 128, Sec. 2, Academia Rd., Nankang, Taipei, TAIWAN, R.O.C.

**Keywords:** Bottom Trawl, Taiwan, IPT, Darwin Core, Fishery

## Abstract

Bottom trawl fishery is one of the most important coastal fisheries in Taiwan both in production and economic values. However, its annual production started to decline due to overfishing since the 1980s. Its bycatch problem also damages the fishery resource seriously. Thus, the government banned the bottom fishery within 3 nautical miles along the shoreline in 1989. To evaluate the effectiveness of this policy, a four year survey was conducted from 2000–2003, in the waters around Taiwan and Penghu (Pescadore) Islands, one region each year respectively. All fish specimens collected from trawling were brought back to lab for identification, individual number count and body weight measurement. These raw data have been integrated and established in Taiwan Fish Database (http://fishdb.sinica.edu.tw). They have also been published through TaiBIF (http://taibif.tw), FishBase and GBIF (website see below). This dataset contains 631 fish species and 3,529 records, making it the most complete demersal fish fauna and their temporal and spatial distributional data on the soft marine habitat in Taiwan.

## Data published through GBIF:

http://fishbase.tw:8080/ipt/resource.do?r=bottom_trawl_survey

## Taxonomic coverage

**“**Fishes of the World” (Nelson 2006) was used as a taxonomic reference for this work.

**Figure 1.**
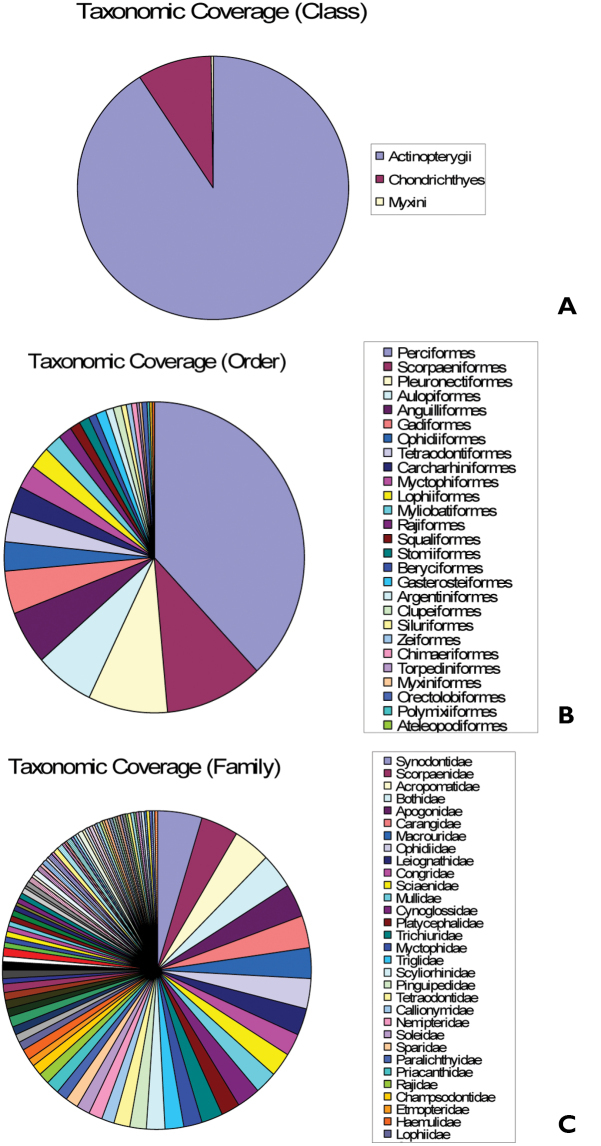
Taxonomic coverage. **A** Class **B** Order **C** Family.

**General taxonomic coverage description:** The coverage (Figure 1) of this dataset includes Class Actinopterygii (90%), Class Chondrichthyes (9%) and Class Myxini (1%).

### Taxonomic ranks

**Kingdom:** Animalia

**Phylum:** Chordata

**Class:** Actinopterygii, Chondrichthyes, Myxini

**Order:** Perciformes, Scorpaeniformes, Pleuronectiformes, Aulopiformes, Anguilliformes, Gadiformes, Ophidiiformes, Tetraodontiformes, Carcharhiniformes, Myctophiformes, Lophiiformes, Myliobatiformes, Rajiformes, Squaliformes, Stomiiformes, Beryciformes, Gasterosteiformes, Argentiniformes, Clupeiformes, Siluriformes, Zeiformes, Chimaeriformes, Torpediniformes, Myxiniformes, Orectolobiformes, Polymixiiformes, Ateleopodiformes, Gonorhynchiformes, Albuliformes, Heterodontiformes, Squatiniformes

**Family:** Perciformes, Scorpaeniformes, Pleuronectiformes, Aulopiformes, Anguilliformes, Gadiformes, Ophidiiformes, Tetraodontiformes, Carcharhiniformes, Myctophiformes, Lophiiformes, Myliobatiformes, Rajiformes, Squaliformes, Stomiiformes, Beryciformes, Gasterosteiformes, Argentiniformes, Clupeiformes, Siluriformes, Zeiformes, Chimaeriformes, Torpediniformes, Myxiniformes, Orectolobiformes, Polymixiiformes, Ateleopodiformes, Gonorhynchiformes, Albuliformes, Heterodontiformes, Squatiniformes

## Spatial coverage

**General spatial coverage:** Seas around Taiwan (Figure 2)

**Coordinates:** 21°25'12"N, 25°40'12"N Latitude; 119°27'36"E, 122°27'36"E Longitude

**Temporal coverage:** February 17, 2000–November 20, 2003

**Figure 2a.**
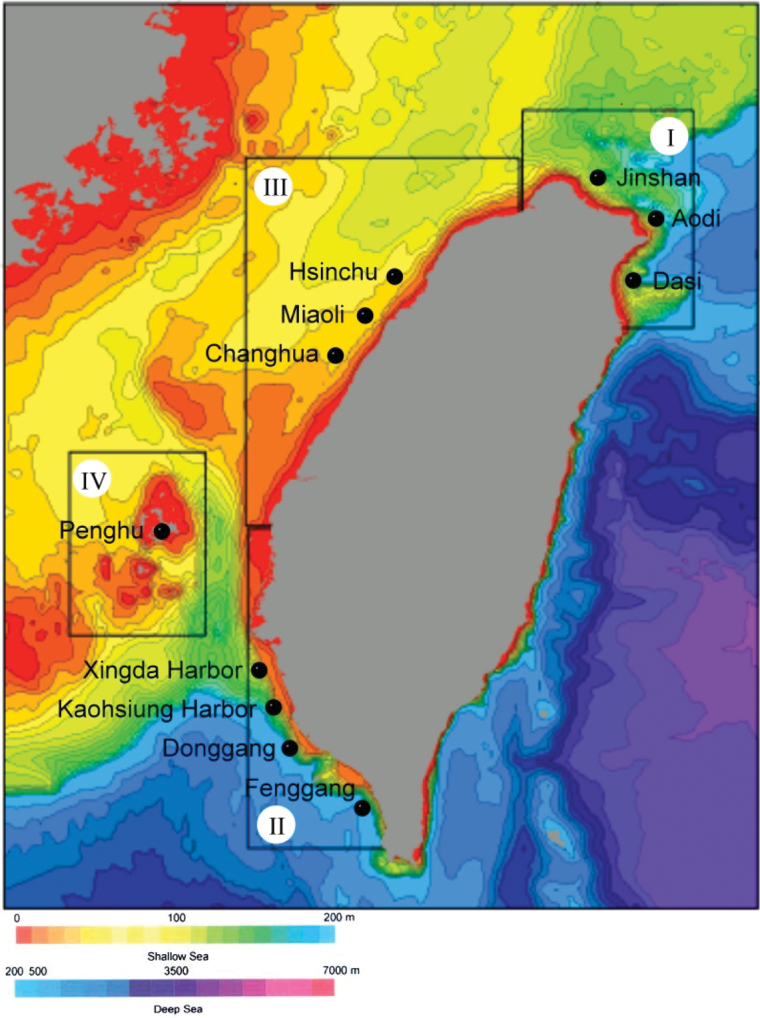
Spatial coverage(fishing harbors of four regions)

**Figure 2b.**
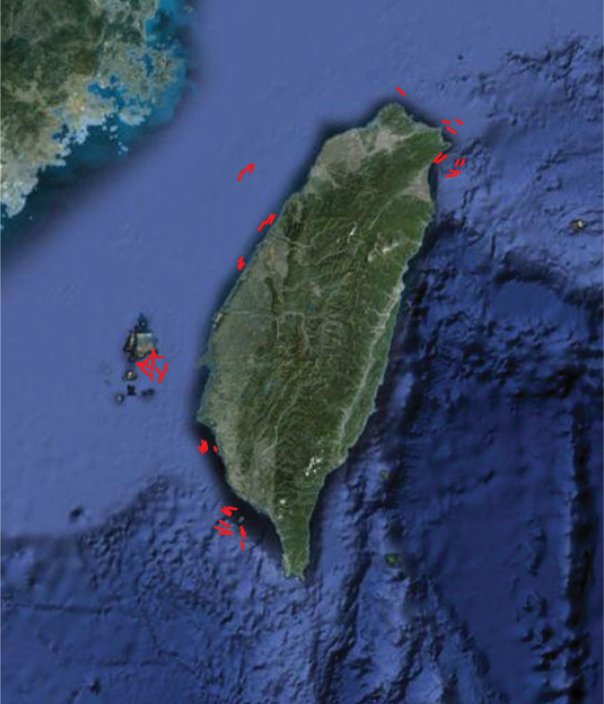
Spatial coverage **(**trawling routes)

## Methods

**Sampling description:** The sampling design for collecting bottom trawl harvest from coastal and offshore waters around Taiwan was to separate the waters into four regions and collect one region each year during 2000–2003 (Figure 2a). In each region except Penghu, 3–4 fixed stations with various water depths were chosen according to different topography and traditional fishing ground in that region. Eastern Taiwan was excluded because it belongs to open ocean and deep-sea environment and there was no bottom trawl fishery in the region. The locality names (or nearest fishing harbours) and water depths in each region were outlined below (Figure 2a and 2b):

2000 – Northern Taiwan (Jinshan: 100 m; mixed with pebbles and sand) and Northeastern Taiwan (Aodi: 100m, 150m and 200 m; and Dasi: 50, 100, 200, 400, 600 and 800 m; all sandy bottoms); 2001 – Southwestern Taiwan (Xingda Harbor: 10, 30, 50, 70 and 100 m; Kaohsiung Harbor: 100, 200 and 300 m; Donggang: 100, 200 and 300 m; Fenggang: 100, 200 and 300 m; sandy and muddy bottoms); 2002 – Western Taiwan (Hsinchu: 60, 65 and 70 m; Miaoli: 10, 30 and 50 m; Changhua: 10, 20 and 40 m; all sandy bottoms); and 2003 – Penghu Islands (16 depths ranging from 25 to 95 m; all sandy and muddy bottoms).

Different bottom trawl fishing boats were used in different regions or harbours with slightly different fishing gears designed for our samplings. Basically at each station, the sampling was with an otter trawl towing at 3 nm/hr for one hour. The longitude and latitude data were recorded using the GPS system on the boat. After the recovery of trawl, all the specimens were identified to the species level, number of individuals for each species counted and body weight measured.

**Quality control description:** All the scientific names of fish samples were validated by the updated fish checklist in our Taiwan Fish Database or TaiBNET (http://taibnet.sinica.edu.tw) before they are added into database. Afterward, they were validated again by matching them against FishBase and Catalog of Fishes, California Academy of Sciences for further correction. If a specimen was rare or it might belong to an undescribed or new species, it was photographed in fresh and then the specimen and its tissue sample were both catalogued and deposited at the Biodiversity Research Museum of Biodiversity Research Center (ASIZP of BRCAS). The latitude and longitude of trawling routes were plotted on Google Maps and outliers detected.

## Project details

**Project title:** Bottom trawl surveys of fishery resources in Taiwan

**Personnel:** Kwang-Tsao Shao (Project Director), Jack Lin (Software enginner and database manager), Chung-Han Wu, Tun-Yuan Cheng, Ruei-Hsien Wu, Jeng-I Tsai, Pai-Lei Lin, Ching-Yi Chen and Hsin-Ming Yeh (field work, fish identification, data collection and analysis)

**Funding:** Council of Agriculture, Executive Yuan, R.O.C. (Taiwan)

**Study area descriptions/descriptor:** This project was carried out for four years from February 2000 to November 2003, one region each year in the following order: (1) off the coast of northern and northeastern Taiwan, (2) coastal waters of southwestern Taiwan, (3) coastal waters of western Taiwan, and (4) Penghu waters (Taiwan Strait). Northern Taiwan stations lie on the borders with China’s continental shelf and the Okinawa Trough. Outside of the northeastern Taiwan stations is the Okinawa Trough. The western Taiwan stations and Pescadores are all on the shallow Taiwan Strait Shelf. The stations in southwestern Taiwan are near the slope of the South China Sea Basin.

**Design description:** Three or four different water depth fixed stations were chosen in each region, except Penghu, for seasonal sampling. In principle, the sampling methods were planned for different locations at different depths of the stations in accordance with sediment, topography, water depth, and the ability of fishing vessel. Sampling was conducted four times a year (quarterly). After sampling, the species composition between different locations, depths, and seasons were compared and analyzed.

## Datasets

**Dataset description:** This project is an outcome of collaborative work with specialists of crustacean (Ming-Shiou Jeng) and mollusks (Chung-Chern Lu). Fish data is the most complete one and the only data which have been open for public access. The dataset includes Station number, locality name, depth, collection date, latitude, longitude, family name, Chinese family name, species name and Chinese name. Because the data also include the number of individuals and biomass range for each species, it could be used for calculating the biodiversity indices, K-dominance (A-B-C) curve, and community structure analysis by using various clustering or ordination methods. If the crustacean and mollusk data can be integrated, an ecosystem trophic model could be elaborated. All data in this database is a good baseline data for the time period of 2000-2003. If the data could be collected again a few years later for comparison, it would be useful to assess whether the enforcement of bottom trawling inside or outside the 3 nautical miles in different regions was effective or not. More detailed analyses of certain dominant species with their body size may also generate some information about their early life history and their inshore or offshore migration or recruitment.

**Object name:** Darwin Core Archive Bottom Trawl Survey

**Character encoding:** UTF-8

**Format name:** Darwin Core Archive format

**Format version:** 1.0

**Distribution:** http://fishbase.tw:8080/ipt/archive.do?r=bottom_trawl_survey

**Publication date of data:** 2011-09-08

**Language:** English

**Metadata language:** English

**Date of metadata creation:** 2011-09-08

**Hierarchy level:** Dataset
